# Quantification of *Phytophthora infestans* population densities and their changes in potato field soil using real-time PCR

**DOI:** 10.1038/s41598-021-85492-z

**Published:** 2021-03-18

**Authors:** Hisashi Osawa, Nobuyuki Suzuki, Seishi Akino, Hiromichi Araki, Kenji Asano, Kotaro Akai, Norio Kondo

**Affiliations:** 1 Graduate School of Agriculture, Hokkaido University, Kita-ku Kita 9 Nishi 9, Sapporo, 060-8589 Japan; 2 Research Faculty of Agriculture, Hokkaido University, Kita-ku Kita 9 Nishi 9, Sapporo, 060-8589 Japan; 3 Potato Research Center, Calbee Potato Inc., 3-23, Minami, Higashimemuro, Memuro, Hokkaido 082-0006 Japan; 4 Hokkaido Agricultural Research Center, NARO, 9-4, Shinseiminami, Memuro, Hokkaido 082-0081 Japan; 5 Present Address: Hokkaido Agricultural Research Center, NARO, 1, Hitsujigaoka, Toyohira-ku, Sapporo, Hokkaido 062-8555 Japan

**Keywords:** Microbial ecology, Microbiology techniques

## Abstract

Tuber infection of *Phytophthora infestans* often occurs at harvest. However, it is difficult to accurately estimate the population densities of *P. infestans* in soil, especially Japanese soil. In the present study, *P. infestans* DNA was extracted from soil samples using a modified CTAB-bead method and quantified using real-time PCR to accurately, rapidly and easily estimate the *P. infestans* population densities in upland soils in Japan. *P. infestans* was well quantified in eleven types of soil samples, including nine types of upland soils in Japan, that were artificially inoculated with a zoosporangia suspension. The amounts of *P. infestans* DNA estimated by the real-time PCR were proportional to the inoculum densities. In the non-controlled experimental potato field, *P. infestans* population densities in soil corresponded to the development of symptoms and were correlated with the number of lesions on the potato foliage. These results imply that the proposed real-time PCR assay is suitable for the estimation or monitoring of *P. infestans* population densities in upland soils in Japan. The population densities at the ridge bottoms were larger than those at any other location in commercial potato fields. These results were similar to those of a previous report using a bioassay. Moreover, a correlation between DNA quantity and inoculum potential was observed. In conclusion, the real-time PCR assay developed in this study is suitable for indirect estimation of the inoculum potential of *P. infestans*.

## Introduction

Various kinds of pathogenic microorganisms cause potato tuber rot; however, tuber rot caused by *Phytophthora infestans* is common in the potato industry as tuber blight. Tuber infection occurs during the growth period^1,2^, at harvest^3^ and handling^4^. Moreover, surface injury at harvest and the presence of *P. infestans* sporangia or blighted plant material are important factors affecting the incidence of potato storage rot in Japanese potato production^5^. Thus, the severity or amount of potato tuber rot in storage facilities can be predicted by estimating the population densities of *P. infestans* in field soils at harvest.

In previous studies, bioassays using healthy leaflet or tuber slices were mainly applied to quantify the population densities of *P. infestans* in soil^2,6,7^. However, bioassays have the disadvantages of requiring (1) equalized conditions and (2) an incubation period. Thus, we applied a real-time PCR assay that can accurately and rapidly provide quantitative results and has a wide dynamic range. DNA extraction from soil can often be difficult, and the addition of skim milk to the extraction buffer has been shown to promote the extraction of DNA from volcanic ash soil^8^. Quantification of the population densities of soil-borne plant pathogens has already been reported^9^, even for *P. infestans*^10,11^. However, naturally infested soils were not quantified in these previous studies; one soil type was usually studied following inoculation with the pathogens. In Japan, volcanic ash soil, such as andosol, is widespread and used to cultivate potatoes, and is known to increase the difficulty of extracting DNA from soil due to the absorption of DNA by soil colloids^8^. Thus, there are no reports on how *P. infestans* population densities in soil fluctuate in actual potato production fields. Additionally, we have to examine whether there are positive correlations between the amounts of DNA quantified by real-time PCR and foliage symptom development, such as the number of lesions.

If this real-time PCR method is established, we will be able to detect inoculum concentrations in sample soils by using real-time PCR instead of a bioassay to accurately predict the risk of disease development. This real-time PCR assay will be applicable to various methods of disease control. For example, the possibilities or severity of potato storage rot may be predicted by estimating the *P. infestans* population density in soil immediately before harvesting. Moreover, we might apply this assay to soil diagnosis before planting. The objectives of this study are (1) to develop a quantitative real-time PCR assay method using various kinds of upland soils in Japan, (2) to estimate the *P. infestans* population densities in potato fields using real-time PCR assay, and (3) to verify the relationship between quantities of *P. infestans* DNA and inoculum potential for further research to reduce potato storage rot in storage facilities.

## Results

### Quantification of the population density of *P. infestans* in inoculated soils

**Figure 1:**
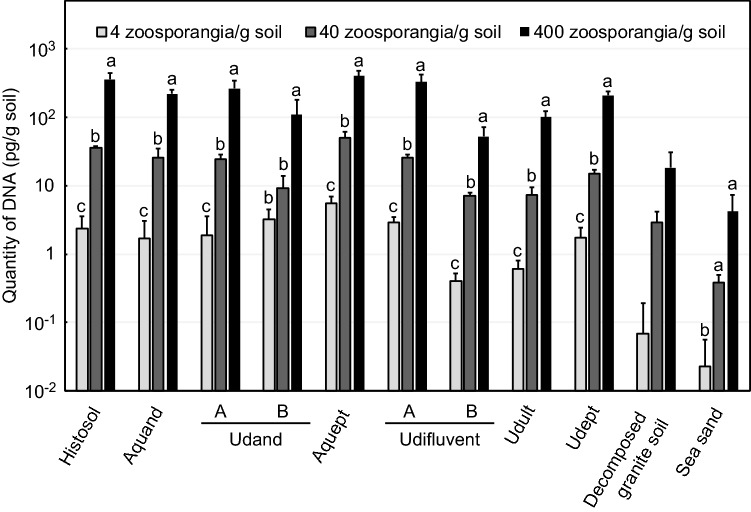
Quantities of *P. infestans* DNA determined using real-time PCR. No *P. infestans* DNA was detected in the negative control plots. Bars indicate the standard deviation. Means (n = 3) with different letters in the same soil type differed significantly according to post hoc tests (*p* < 0.05, Tukey’s test, Games-Howell test or Steel–Dwass test).

From each of the 11 soil types inoculated with the pathogen, DNA was detected in proportion to the densities of the inoculum, while no amplification was found in negative control samples (Fig. 1, Supplementary Fig. S1 online) as well as in a preliminary experiment^12^. Even low densities in soil samples containing approximately 4 zoosporangia/g soil (2 zoosporangia/tube) were well quantified; the quantities of DNA were approximately 1 pg/g soil (10 fg/µL). However, in decomposed granite soil and sea sand, smaller amounts of DNA were quantified. Additionally, in udifluvent B and an udult, slightly smaller amounts of DNA were quantified than in any other upland soils. However, almost the same amounts of DNA were quantified if soils contained the same numbers of *P. infestans* zoosporangia.

The DNA quantities were reflected in the population densities. When the inoculum density was 10 times higher, approximately 10 times more DNA was detected. All soil types showed significant differences according to one-way analyses of variance (ANOVA) (histosol, aquand, udand A, udand B, aquept, udifluvent A, udifluvent B, udult and udept: *p* < 0.01; sea sand: *p* = 0.01) or Kruskal–Wallis test (decomposed granite soil: *p* = 0.03). Moreover, according to post hoc tests, the mean amount of *P. infestans* DNA detected in each soil type was significantly different except for that observed in some udand B, decomposed granite soil and sea sand samples (Fig. 1).

### Changes in *P. infestans* population densities in non-controlled field soil

We observed symptom development and analysed two types of soils samples, from ridgetops and peripheral areas, in noncontrolled potato fields in the Hokkaido Agricultural Research Center (HARC) during the cultivation period in 2017 and 2018.

**Figure 2:**
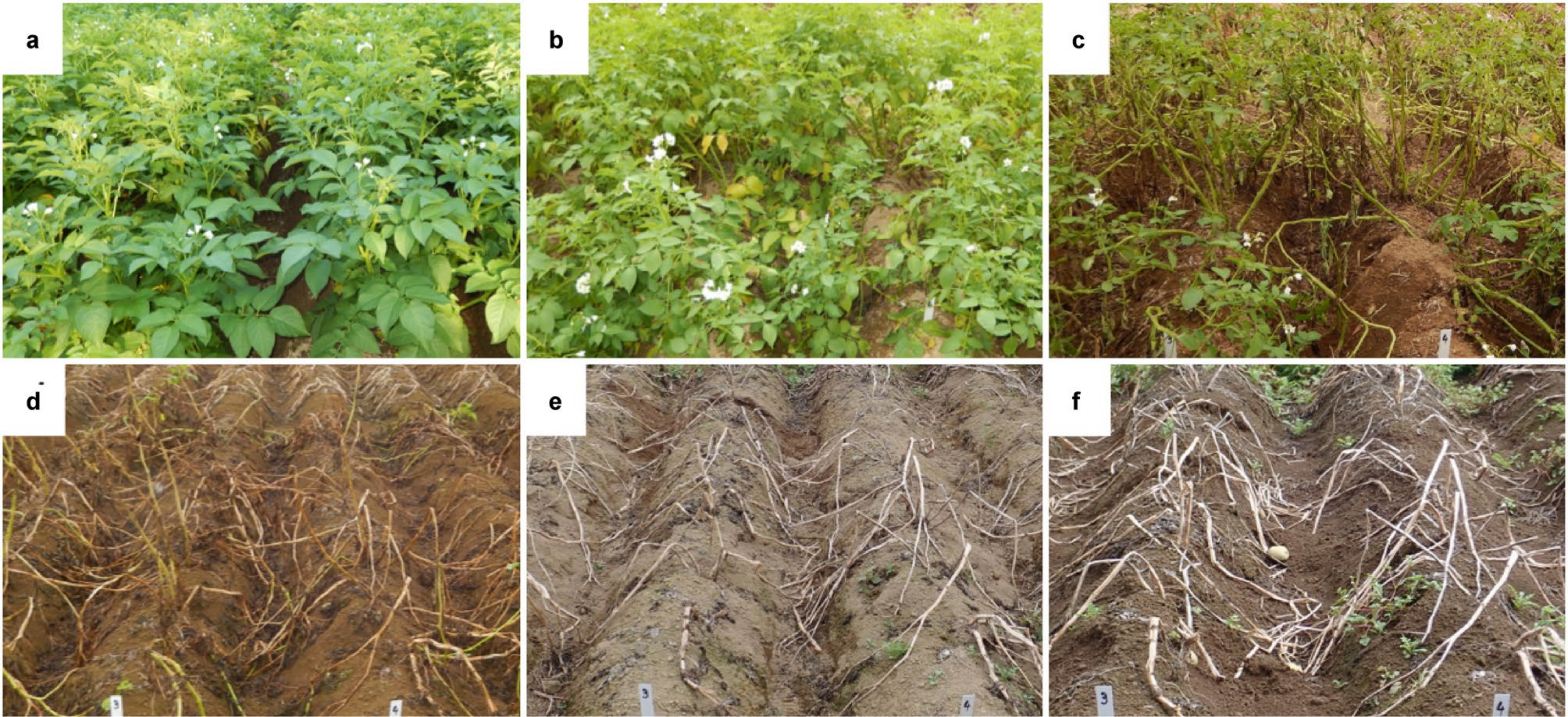
Symptom development at the same place in a non-controlled potato field in 2017. (**a**) First observation of late blight in the field on 7 July. (**b**) First observation of late blight on the sampled plants on 28 July. **c** An epidemic occurred on 5 August. (**d**) All plants died of late blight by 18 August. (**e**) The plants were dried by 25 August. (**f**) The final day of sampling on 15 September.

**Figure 3:**
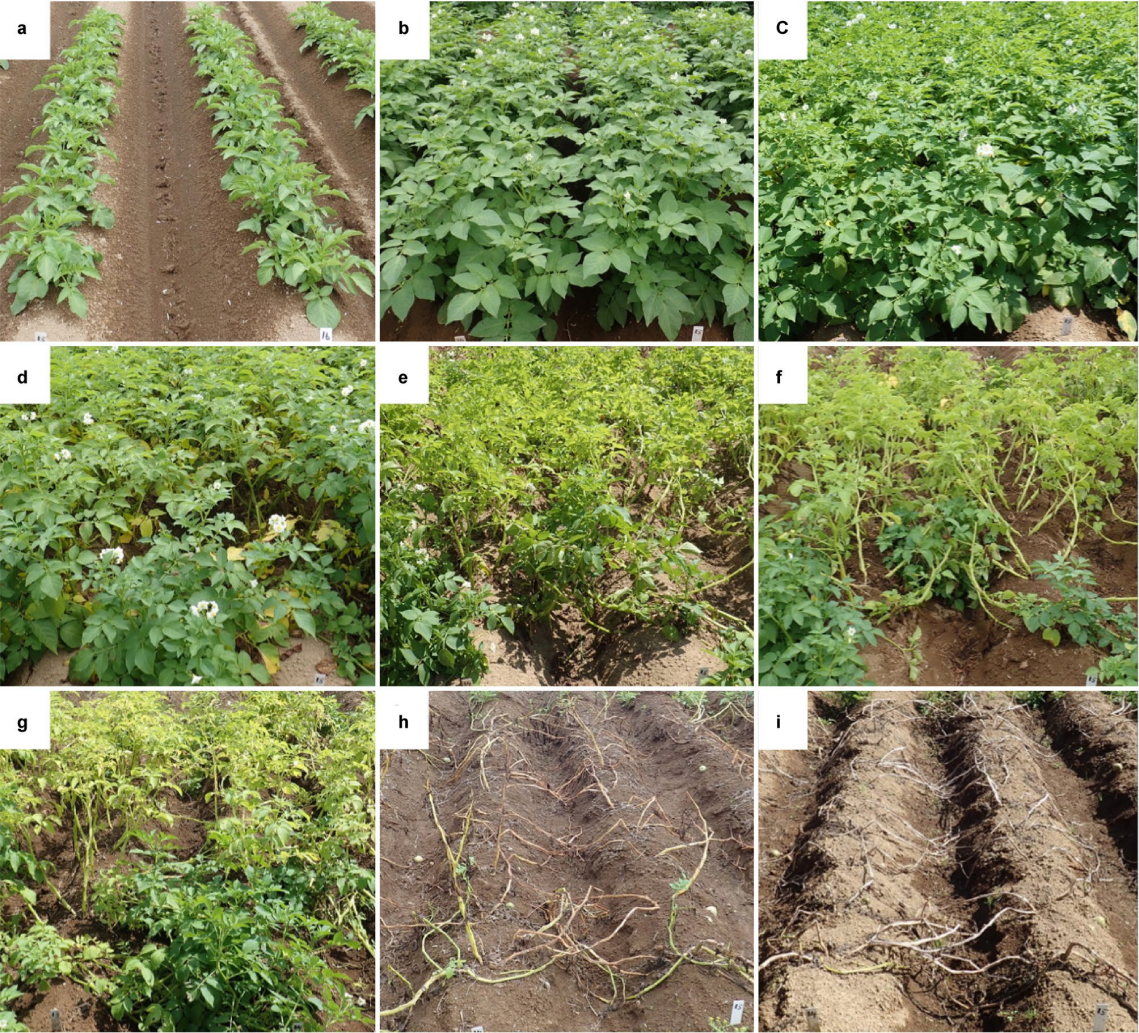
Symptom development at the same place in a non-controlled potato field in 2018. (**a**) Before the first observation of late blight in a field on 15 June. (**b**) First observation of late blight in the field on 6 July. (**c**) First observation of late blight on the sampled plants on 20 July. (**d**) The number of lesions increased on 27 July. (**e**) The number of lesions decreased due to a heat wave on 3 August. (**f**) Most infested leaves were dried and senesced by 25 August. (**g**) An epidemic occurred on 18 August. (**h)** All plants died of late blight by 31 August. (**i**) The plants were dried by 25 August.

In 2017, late blight occurred in the field on 7 July, and some lesions were found for the first time at a sampling location on 28 July. After that, an epidemic occurred on 5 August, and all of the plants had died of late blight by 18 August. The plants were dried by 25 August (Fig. 2). In 2018, late blight was first observed in the field on 6 July and on a sampled plant on 20 July. On 27 July, there were 21.7 lesions/plant; however, the number of lesions on the foliage was decreased due to a heat wave such that only 6 lesions/plant occurred on 5 August. The heat wave continued for a while, and a second epidemic occurred on 18 August. After the second epidemic, all plants had died by 31 August (Fig. 3). Moreover, a positive correlation (*r* = 0.41, *p* = 0.03) was observed between the quantity of *P. infestans* DNA and the number of lesions.

**Figure 4:**
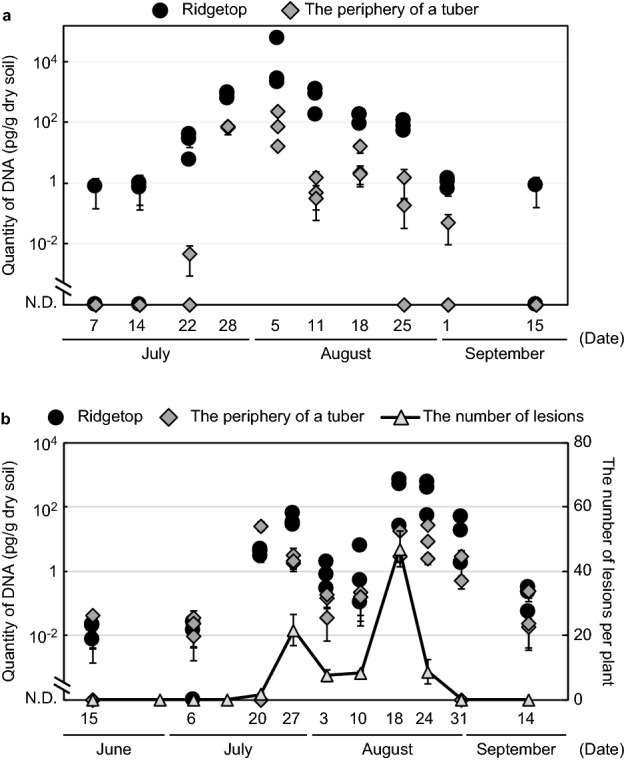
Changes in the quantities of *P. infestans* DNA and the number of foliage lesions. N.D. means not detected. Bars indicate the standard error. (**a**) 2017 results. (**b**) 2018 results.

The quantities of *P. infestans* DNA were small before the outbreak of late blight and when late blight was first observed in the field both in 2017 and 2018. Next, the amounts of DNA increased with foliage symptom development. The amount of DNA in ridgetop soils was 10^4^–10^5^ times larger during the epidemic than when the disease was first observed. After the epidemic, the amounts of DNA decreased with the death and drying of the foliage. There was a small amount of DNA in the ridgetop soil one month after foliage drying in 2017. Most ridgetop samples had larger amounts of DNA than the soil samples from the tuber periphery. This phenomenon was remarkable in 2017 (Fig. 4). Obviously blighted tubers, however, were not found in the sampling sites.

### Quantities of *P. infestans* DNA and inoculum potential in commercial field soils

**Figure 5:**
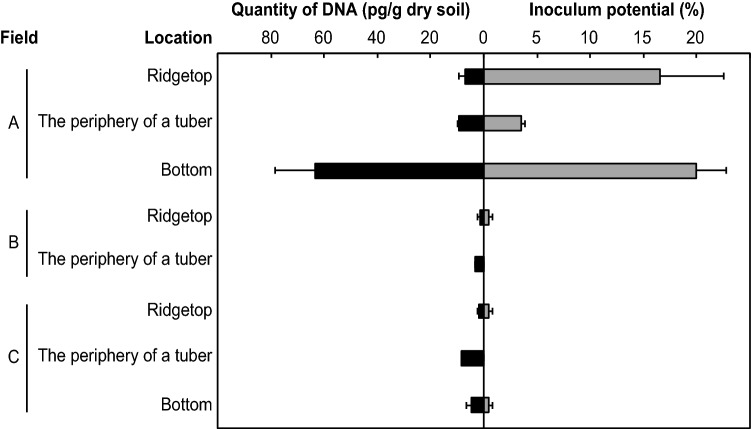
Quantities of *P. infestans* DNA and inoculum potential from soils in commercial fields. Bars indicate the standard error. Fields A, B, and C indicate separate potato fields in the Tokachi region, Hokkaido, Japan.

The quantities of DNA were larger in field A than in fields B or C. Soil samples from ridge bottoms contained especially large amounts of DNA. In field A, inoculum potentials were also large, while field B or C showed smaller inoculum potentials than field A (Fig. 5). Moreover, a positive correlation (*r* = 0.88, *p* = 0.02) was observed between the quantity of *P. infestans* DNA and inoculum potential.

The amount of DNA quantified in the tuber periphery soil samples was larger than that in ridgetop soil samples. These results were different from the results of the non-controlled field tests described above. However, the inoculum potentials of the tuber periphery samples were smaller than those of ridgetop samples. Blighted tubers were not found in the sampling sites.

## Discussion

We modified the reported DNA extraction methods using a commercial DNA extraction kit: the cetyl trimethylammonium bromide (CTAB) method^13^ with the addition of skim milk to prevent the absorption of DNA and a bead beating method^14^. In this report, this method is named the modified CTAB-bead method. The proposed real-time PCR assay may be suitable for the quantification of *P. infestans* population densities, at least in Japanese upland soils, because *P. infestans* DNA from various kinds of upland soils was well quantified, and there were no false positives in the negative control plots. Thus, we conclude that the *P. infestans* population density can be represented by the quantity of DNA determined using real-time PCR. One udifluvent and udult soil quantified slightly small amounts of DNA, and there were small differences among soil types at the same population densities. However, this should not be of great consequence because the differences compared with the other upland soils are within tenfold; thus, these small differences are likely due to the soil characteristics. A previous study reported that no single method of cell lysis or purification is appropriate for all soils^15^. Thus, the proposed real-time PCR assay is available to quantify the pathogen densities in soils such that most soil samples containing 4–400 zoosporangia/g soil plots except decomposed granite soil and sea sand were quantified as approximately 1–100 pg/g soil. Although this method can be used to quantify *P. infestans* DNA levels in soil, not all soil samples containing the same number of zoosporangia yielded similar results, as the amount of DNA absorbed was dependent on the soil type. Thus, a calibration curve may be required when a new soil type is tested in which a zoosporangia suspension or *P. infestans* DNA is added to nondiseased soil. Regarding decomposed granite soil and sea sand, which are not upland soils and not suitable for potato cultivation, the reason for the small DNA quantities may be that a large amount of DNA is absorbed onto silica under Na^+^- or Ca^2+^-rich conditions^16^. If the soil type is sandy or clayey, the DNA quantities may be smaller than those in other soil types. For further development of this method, the addition of an internal control, such as GFP-induced plasmid DNA^17^, to correct the raw data might be effective. Additionally, changing the glass beads used in this method to zirconia or iron beads may also be effective due to the powerful homogenization and lower amount of DNA absorption achieved with the latter two bead types. However, these improvements may be unnecessary because the proposed assay has a small detection limit such that samples containing only 4 zoosporangia/g soil were detected and quantified. Ristaino et al.^18^ reported that real-time LAMP and droplet digital PCR can be used to quantify *P. infestans* DNA from plant tissue. Compared with these tools, the proposed real-time PCR assay has some advantages, such as a wide dynamic range. For this reason, this assay may be widely applied to upland soils.

This is the first report of the quantification of *P. infestans* population densities in naturally infested soil samples, and changes in the population densities were analysed using real-time PCR. These results also showed that this quantitative method provides reproducible results, because changes in *P. infestans* DNA were correlated with symptom development throughout the growth periods. DNA quantities during the epidemics (5 and 18 August 2017 and 2018, respectively) were converted into *P. infestans* population densities in zoosporangia equivalents based on the results obtained for udant B (experimental field, HARC), as shown in Fig. 1; thus, there were approximately 10^4^–10^5^ and 10^3^–10^4^ zoosporangia in the ridgetop soils. These results indicate that a large amount of *P. infestans* existed in the field ridgetop soils where the plants were blighted. Quantified DNA may be from zoosporangia, mycelia, or small residue or free DNA but not from oospores. Because the A1 mating type has been dominant in Japan since 2005^19^, sexual reproduction rarely occurs, at least in potato fields. We have not verified the availability of soils containing oospores. In future studies, inoculated soil containing oospores should be tested. However, soils containing *P. infestans* oospores might be quantified using the proposed assay because previous studies reported that soils containing three potato pathogens and oospores of *Pythium* spp. have been quantified using CTAB and bead beating methods^20,21^. If the proposed assay can quantify *P. infestans* DNA from oospores in soil, we might apply this assay to soil diagnosis before planting.

A previous study reported that the inoculum potentials of soil decreased as foliage lesions became less abundant^2^. Our study corresponds to this previous study because the quantities of *P. infestans* DNA in soil were consistent with foliage symptom development (in 2017 and 2018) and the number of lesions per plant (in 2018). Hence, the proposed real-time PCR method can be an alternative to bioassays and used as a method to quantify the *P. infestans* population density. Bioassays require special knowledge and techniques of plant pathology because researchers have to judge whether inoculated tubers were rotted due to *P. infestans*. On the other hand, real-time PCR assays are easy and require only minimal knowledge and techniques of molecular biology. In 2018, symptom development stopped from late July to early August due to a heat wave. The DNA quantities were reflected in foliage symptoms, with small quantities of DNA estimated during this period. These results imply that this method is highly sensitive for estimating even weekly population changes. The quantities of DNA were decreased to one-tenth of their former numbers in a week after the desiccation of the foliage. As indicated by the decrease, most *P. infestans* zoospores or zoosporangia cannot survive in/on the soil and quickly die and are degraded by microorganisms and DNase^6,22,23^. However, if new A2 strains migrated into Japan and oospores were found in field soil, DNA would be detected for a long time even in the noncultivation period. Surprisingly, an infinitesimal quantity of DNA was detected one month after the foliage had disappeared in 2017. This DNA may have been from another plant out of experimental fields or DNA absorbed to some kind of soil material and may persist against DNase^24^.

Figure 5 shows a positive correlation between the quantity of *P. infestans* DNA and the inoculum potential. Thus, the proposed real-time PCR method is suitable for indirect estimation of *P. infestans* inoculum potential. In this analysis, two data sets containing zero values were eliminated as outliers because a zero value in this experiment signifies “below the detection limit”; we cannot determine the exact value. Thus, data sets containing zero values cannot be included in the analysis to evaluate the applicability of the proposed real-time PCR assay instead of the bioassay for estimating inoculum potential. Previous estimation methods, such as bioassays, require an incubation period of approximately 2–3 weeks, expert knowledge of *P. infestans* and incubation space. On the other hand, real-time PCR requires several hours to estimate the population densities, minimal knowledge of molecular biology and no incubation space. For these reasons, we can more easily estimate *P. infestans* inoculum potential with real-time PCR than with bioassays. In the experiments using commercial potato fields, a larger amount of DNA was quantified from ridge bottom soils than from any other location. This result agrees with a previous report that most rainwater was deposited at the bottoms of ridges, and the rainwater contained fewer than 500 zoosporangia when blight was present on the crop^25^. According to this result, soils sampled from the bottom of a ridge are suitable for whole field estimation of *P. infestans* population densities.

In this study, the quantities of DNA and inoculum potentials were larger in field A than in fields B and C. This result suggests that the proposed real-time PCR assay may be suitable for comparison among potato fields. In field A, late blight occurred because farmers could not conduct chemical control due to heavy rain. Field B was a non-controlled commercial field, and incomplete chemical spraying gave rise to a non-controlled spot in field C. Fields B and C were not perfectly managed for preventing late blight; however, some control, such as cultural or chemical control, was performed in some part. On the other hand, field A did not receive control measures at all. This might be why field A had larger DNA quantities and inoculum potentials than fields B and C.

For the results from non-controlled fields, *P. infestans* did not percolate through the soil but instead remained at the surface because most soil samples from ridgetops contained larger amounts of DNA than those from the tuber periphery. A previous study reported that more than half of the tubers in the top 5.1 cm of soil were blighted, and the population of blighted tubers decreased with increasing depth^26^. We can show the same conclusions using real-time PCR. However, in commercial fields, all soils sampled from the tuber periphery contained larger amounts of DNA and had a lower inoculum potential than those from the ridge surfaces. Rainy and cold conditions (approximately at 13 °C) continued from 14 to 18 August 2018, several days before sampling^27^. The weather might have sustained indirect germination, and many zoospores were released and percolated through the soil. However, zoospores are motile for only a short time^28^ and cannot survive for a long time. Thus, much of the quantified DNA was from dead *P. infestans* or free DNA, and less inoculum potential was found near tubers. Soil samples from ridgetops showed larger inoculum potential than those from the tuber periphery. This may be because ridgetop samples contain a large amount of fresh *P. infestans* from foliage lesions.

In this study, we successfully developed a real-time PCR assay to estimate the *P. infestans* densities in upland soils, and the proposed assay is available not only for the estimation of population density but also inoculum potential. In the future, this research can provide to a new decision support system for predicting and preventing potato storage rot. The *P. infestans* soil population density is the most important factor influencing potato storage rot. The possibilities or severities of potato storage rot may be predicted by estimating the *P. infestans* population density in soil before harvesting. Previous storage planning suggested that potato storage rot might occur if many foliage lesions occur during the growing season. In this study, most *P. infestans* DNA from foliage lesions degraded within one week. Thus, the possibility and severity of storage rot may be low if the quantity of *P. infestans* DNA immediately before harvesting is small, even if many foliage lesions occur during the growing season. Additionally, the previous quantitative method (bioassay) requires an incubation period of approximately one week or more^2,7^. On the other hand, the real-time PCR assay does not require an incubation period, and it takes only several hours to quantify the *P. infestans* population density in the sample soil. Potato storage rot may be reduced because the storage plan can be selected accurately and rapidly by using real-time PCR compared with previous methods. For example, tubers harvested from fields harbouring high levels of *P. infestans* DNA can be shipped as soon as possible to prevent potato storage rot. However, many other factors may be involved in the spread of this disease, such as surface injury^5^. A decision support system would allow potato storage companies to evaluate and address factors associated with potato storage rot and establish appropriate countermeasures to prevent economic losses.

## Methods

### DNA extraction from inoculated soil

First, ϕ 0.5 mm (ASONE) and ϕ 0.1 mm (Sigma-Aldrich) glass beads were washed with hydrochloric acid and dried at 180 °C for 2 h. Then, 0.4 g of each of 11 types of healthy soils (Supplementary Table 1 online) was added to tubes containing 0.2 g of ϕ 0.5 mm glass beads and 0.1 g of ϕ 0.1 mm glass beads. A *P. infestans* isolate (MR1799: available from NARO Genebank, Japan as MAFF247164) was incubated on rye-B agar medium^29^ at 15 °C for 2 weeks to make a zoosporangia suspension. Zoosporangia suspensions were adjusted to 2.3 × 10^3^, 2.3 × 10^2^, and 2.3 × 10^1^ zoosporangia/mL with distilled water. First, 0.5 g of artificially inoculated soils (approximately 400, 40 and 4 zoosporangia/g soil) was made by adding 100 µL of these zoosporangia suspensions to 0.4 g of soil in a tube. In negative control plots, distilled water was added instead of zoosporangia suspension. Inoculated soils in bead tubes were stored at − 20 °C, and all plots were triplicated.

DNA was extracted from soil samples collected in Japan using a NucleoSpin Plant II kit (Macherey–Nagel) with a modified extraction buffer (2 M NaCl, 20 mM EDTA-2Na, 100 mM Tris–HCl (pH 8.0), 2% CTAB, 2% polyvinylpyrrolidone K-30, 12.5% skim milk, and 7 mg/L of salmon sperm DNA) following the manufacturer’s protocol and that of Sato et al.^30^. Then, 800 µL of the extraction buffer was added to the bead tube, and the tubes were heated in a microwave oven (200 W, 10 s, three times). After heating, the samples were homogenized twice at 4.0 m/s for 20 s (FastPrep 24 Instrument, MP Biomedicals). Homogenized samples were centrifuged for 10 min at 5000 g at 20 °C. The obtained clear supernatant was purified based on the kit protocol. Finally, a total of 50 µL of DNA template per sample was obtained and stored at − 20 °C.

### Real-time PCR

Real-time PCR was carried out using a StepOnePlus Real-time PCR system (Applied Biosystems) and Probe qPCR Mix (Takara Bio). The reaction mixture was made in a final volume of 20 µL containing 10 µL of Probe qPCR mix, 0.3 M of each primer^10^, 0.1 M TaqMan probe^10^, 0.2 M ROX reference dye and 2 µL of template DNA. PCR was carried out under the recommended conditions (95 °C for 30 s, (95 °C for 5 s and 61 °C for 30 s) × 45 cycles). As a negative control, distilled water was used instead of template DNA. The genomic DNA of *P. infestans* was adjusted to 0.01, 0.1, 1, 10, 100 and 1000 pg/µL TE using a spectrophotometer (BioSpec-nano, Shimadzu) and used as standards. The threshold was determined automatically by StepOne software v.2.1.

### Analyses of changes in the *P. infestans* population densities in non-controlled fields

Soil samples were obtained in a non-controlled potato field (cv. Snowden, a typical cultivar in the Tokachi region and known to be susceptible to leaf and tuber blight) (udand) in HARC, Japan, during the cultivation period (July to September) in 2017 and 2018 (Supplementary Table 1 online). Three plants were randomly selected and sampled along with a handful of ridgetop soil. Next, soils from the periphery of tubers (approximately 15 cm depth from ridgetop) were sampled in the same manner after removing ridgetop soil to prevent contamination of ridgetop soil in the samples. Each sample was mixed evenly and stored at − 20 °C until DNA extraction. DNA extraction and quantification were carried out using 0.5 g of soil sample (n = 3) in the same method described above. The soil moisture contents were also investigated by drying at 105 °C for 24 h. In 2018, the number of foliage lesions on sampled plants was counted (n = 3).

### Quantification of population densities and inoculum potentials from soils in commercial fields

Soils were sampled in the commercial potato fields A, B and C, where late blight was observed in the Tokachi region, Hokkaido, Japan on 20 August 2018. Soils were sampled from the area around five plants evenly distributed throughout the diseased area and mixed in approximately equal amounts. Locations of soil samples were the same as described above: ridgetops and tuber peripheries. Additionally, surface soils from the bottom of a ridge were sampled in the same manner in fields A and C. After sampling, soils were divided into two groups: one group was used for quantification of the population densities using real-time PCR, and the other group was used for quantification of inoculum potentials using the bioassay described in Sato^2^. Soil samples for real-time PCR were stored at − 20 °C as soon as possible. The bioassay was carried out on 22 August 2018. After incubation, the number of rotten tuber pieces with *P. infestans* mycelia and zoosporangia observed by stereoscopic microscopy were counted among 100 total pieces per plot. The inoculum potential equation used in this study was as follows: inoculum potential (%) = (the number of rotten tuber pieces)/(the number of total tuber pieces) × 100. DNA extraction and real-time PCR were carried out in the same manner described above. Two and three replicates were performed for the bioassay and real-time PCR, respectively.

### Statistical analyses

Before statistical analyses, quantities of *P. infestans* DNA were log-transformed, and their normality and equality of variance were checked by Shapiro–Wilk test and Bartlett’s test, respectively. To compare the quantity of *P. infestans* DNA in artificially inoculated soil samples, ANOVA was applied for each soil type except for decomposed granite soil and sea sand. For decomposed granite soil, the Kruskal–Wallis test was used instead of ANOVA because the dependent variables were not normally distributed. For sea sand, Welch’s ANOVA was employed because the data did not have equality of variance. When the ANOVA result was significant (*p* < 0.05), a post hoc test (Tukey’s test) was carried out for each plot except for decomposed granite soil and sea sand. For decomposed granite soil and sea sand, the Steel–Dwass test or Games-Howell test was conducted, respectively.

In the present study, Spearman’s rank correlation coefficients were determined to assess correlations between variables. To determine the correlation between inoculum potential and quantity of DNA, data sets including a zero value were eliminated as outliers.

All statistical analyses were conducted using R software version 3.6.1 and base packages^31^.

## Supplementary Information

Supplementary Information 1.

## Data Availability

All data generated or analysed during this study are included in this published article (and its Supplementary Information files).
