# Supplementary material for: Quantification of Phytophthora infestans population densities and their changes in potato field soil using real-time PCR
Source: Sci Rep. 2021 Mar 18;11:6266. doi: 10.1038/s41598-021-85492-z (PMC7973439; doi:10.1038/s41598-021-85492-z)
Supplement: Supplementary file 1 — Supplementary Information 1. [file 41598_2021_85492_MOESM1_ESM.pdf]

Quantification of *Phytophthora infestans* population densities and their changes in potato field soil using real-time PCR

Hisashi Osawa[1,2,*], Nobuyuki Suzuki[1], Seishi Akino[3,*], Hiromichi Araki[4], Kenji Asano[5], Kotaro Akai[5], and Norio Kondo[3]

[1]Graduate School of Agriculture, Hokkaido University, Kita-ku Kita 9 Nishi 9, Sapporo, 060-8589, Japan

[2] Present address: Hokkaido Agricultural Research Center, NARO, 1, Hitsujigaoka, Toyohira-ku, Sapporo, Hokkaido, 062-8555, Japan

[3]Research Faculty of Agriculture, Hokkaido University, Kita-ku Kita 9 Nishi 9, Sapporo, 060-8589, Japan

[4]Potato Research Center, Calbee Potato Inc., 3-23, Minami, Higashimemuro, Memuro, Hokkaido, 082-0006, Japan

[5]Hokkaido Agricultural Research Center, NARO, 9-4, Shinseiminami, Memuro, Hokkaido, 082-0081, Japan

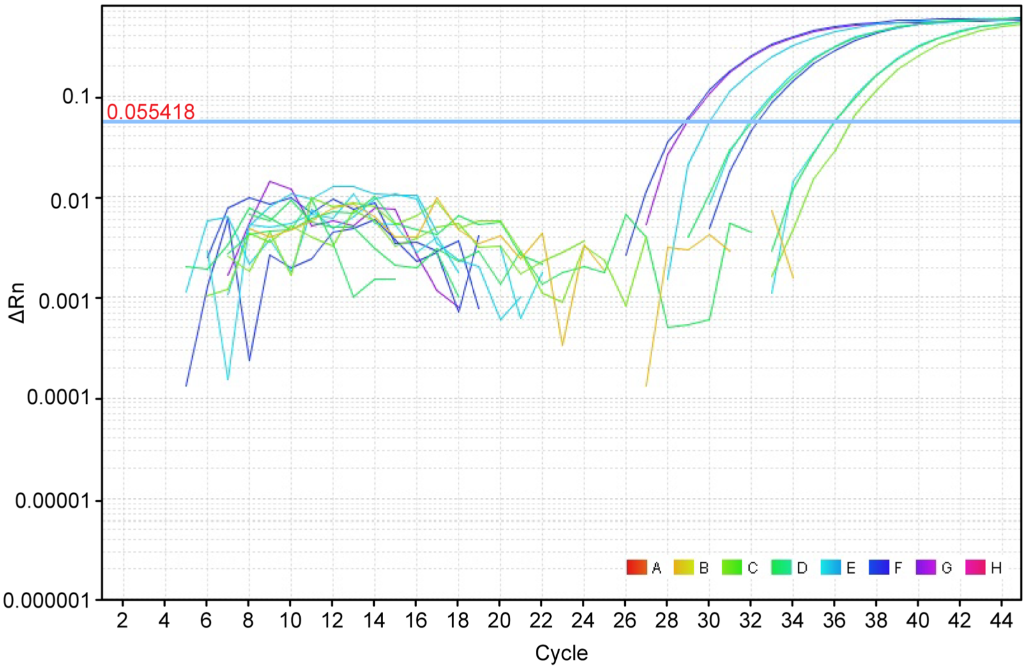

Supplemental Fig. 1. Example of DNA quantification from inoculated soils (udifluvent B).

Supplemental Table 1. Tested soil types.

ID	Soil type	Sampling region	Remarks
1	Histosol	Tokachi, Hokakido, Japan	Upland soil
2	Aquand	Tokachi, Hokakido, Japan	Upland soil
3	Udand A	Tokachi, Hokakido, Japan	Upland soil
4	Udand B	Tokachi, Hokakido, Japan	Upland soil
5	Aquept	Tokachi, Hokakido, Japan	Upland soil
6	Udifluvent A	Tokachi, Hokakido, Japan	Upland soil
7	Udifluvent B	Sapporo, Hokakido, Japan	Upland soil
8	Udult	Kamikawa, Hokkaido, Japan	Upland soil
9	Udept	Kamikawa, Hokkaido, Japan	Upland soil
10	Decomposed granite soil	Awaji, Hyogo, Japan	Forestry soil (Daisei Co. Ltd.)
11	Sea sand (850-1400 µm)	-	For chemical (Wako)
